# The impact of mismatch repair status and systemic inflammatory markers on radiological staging in colon cancer

**DOI:** 10.1259/bjr.20230098

**Published:** 2023-07-26

**Authors:** James R Platt, Jennifer Ansett, Jenny F Seligmann, Nicholas P West, Damian J M Tolan

**Affiliations:** 1 Division of Oncology, Leeds Institute of Medical Research at St James’s, University of Leeds, Leeds, United Kingdom; 2 Department of Cellular Pathology, St James’s University Hospital, Leeds Teaching Hospitals NHS Trust, Leeds, United Kingdom; 3 Division of Pathology and Data Analytics, Leeds Institute of Medical Research at St James’s, University of Leeds, Leeds, United Kingdom; 4 Department of Radiology, St James’s University Hospital, Leeds Teaching Hospitals NHS Trust, Leeds, United Kingdom

## Abstract

**Objective:**

Mismatch repair (MMR) deficient (dMMR) colon cancer (CC) is distinct from MMR proficient (pMMR) CC, yet the impact of MMR status on radiological staging is unclear. The purpose of this study was to investigate how MMR status impacts CC CT staging.

**Methods:**

We retrospectively compared CT staging accuracy between dMMR and pMMR CC patients undergoing curative resection. Accuracy was assessed as individual tumour (T)/nodal (N) stages and as dichotomous “statuses” (T1/2 *vs* T3/4; N0 *vs* N1/2). Patient characteristics were analysed for factors to support staging.

**Results:**

There was no significant difference in overall staging accuracy between the dMMR (44 patients) and pMMR (57 patients) groups. dMMR tumours with incorrect N stage/“status” were more likely to be overstaged than pMMR tumours (90% *vs* 59%; *p* = 0.023 for “N status”). Platelet count, CRP and neutrophil count (AUC 0.76 (*p* = 0.0078), 0.75 (*p* = 0.034) and 0.70 (*p* = 0.044), respectively) were associated with “N status” in dMMR tumours.

**Conclusion:**

Whilst overall staging accuracy was similar between groups, incorrectly N staged dMMR tumours were more likely to be overstaged than pMMR tumours, risking inappropriate surgical or neoadjuvant treatment. We describe novel relationships between several inflammatory markers and pathological “N status” in dMMR CC, which if integrated into routine practice may improve CT staging accuracy.

**Advances in knowledge:**

Compared to pMMR CC, dMMR CC is at significant risk of N overstaging. Platelet count, CRP and neutrophil count are higher in dMMR CC patients with nodal metastases than those without, and their role in refining clinical staging requires further investigation.

## Introduction

Colon cancer (CC) is the fourth most common cancer in the UK, with over 33,000 new cases and over 12,000 deaths each year.^1^ Following diagnosis, radiological staging with CT is used to guide treatment decisions.^2^ The emergence of neoadjuvant chemotherapy (NAC) as an effective treatment for CC has placed greater importance on radiological staging in this setting.^3^

The accuracy of radiological staging in CC has been well studied.^4,5^ Whilst CT is able to achieve reasonable accuracy in predicting primary tumour (T) stage, it performs poorly when predicting lymph node (N) stage. Improving the accuracy of radiological N staging has therefore been the focus of much research, with various radiological parameters investigated; lymph node long diameter >10 mm and a cluster of three or more nodes irrespective of size are the most common. However, the existence of micrometastases in smaller nodes and the presence of enlarged and benign inflammatory nodes have rendered simple size criteria insufficient.^6,7^ Recent studies have shown internal heterogeneity and an irregular outer border to be superior to size criteria for N staging; however, these criteria are more challenging to assess and provide limited improvement in performance.^8,9^

Mismatch repair (MMR) deficiency is a genetic abnormality which arises due to the loss of one or more of the MMR proteins and increases the risk of developing several cancer types, including colorectal, endometrial and ovarian cancers.^10^ MMR deficient (dMMR) CC accounts for 15–20% of CCs^11^ and is distinct from MMR proficient (pMMR) CC with respect to various characteristics. dMMR CC is more common in older, female adults, and is associated with the development of right-sided, mucinous and poorly differentiated tumours. MMR status also influences treatment, with dMMR CC less responsive to conventional chemotherapy but highly sensitive to immunotherapy.^10,12^ Yet, the impact of MMR status on radiological staging has received little attention. The primary objective of this study was to investigate whether the accuracy of radiological staging differs between patients with dMMR and pMMR CC. Our secondary objective was to explore whether certain patient characteristics were associated with pathological staging and thus could be used to support radiological staging.

## Methods

### Study design

This is a retrospective analysis comparing the accuracy of radiological T and N staging between patients with dMMR and pMMR CC. Radiological staging data from the point of diagnosis were used to evaluate real-world clinical practice. Cases were not restaged retrospectively. In view of the retrospective evaluation of routine and anonymised patient data, written consent was not required, and approval for the project was obtained from the local Information Governance Team.

### Patient selection

Patients were eligible for inclusion if they had a histological diagnosis of CC, documented radiological staging, post-operative pathological staging (according to TNM version 8^13^; T1 to T4, N0 to N2 and M0 to M1), and MMR status (obtained by four protein immunohistochemistry). Patients were excluded if they had a non-colonic malignancy (including rectal cancer), synchronous CC, histological subtype other than adenocarcinoma or mucinous adenocarcinoma, active appendicitis, diverticulitis, colitis or perforation when undergoing radiological staging, surgery performed with only palliative intent, prior neoadjuvant treatment, or where radiological staging, pathological staging or MMR status were not accessible within the medical record.

All CT scans were performed on a range of different 128 detector CT scanners with maximum 2.5 mm axial and 3 mm coronal reconstructed slice thickness. Portal venous phase assessment of the abdomen and pelvis was acquired using a weight-based regimen with 350 mg ml^−1^ iodinated contrast and 65 s delay. Cases with non-contrast CT were also included if clinical TNM staging was provided by the reporting radiologist.

All included patients underwent curative surgical resection between 29 March 2019 and 3 July 2020. All eligible patients with dMMR CC from this time period were included. For comparison, a similarly sized representative sample of pMMR CC patients were selected from a total of 339 patients during the same time period. Patients in this group were selected using a random number generator and then assessed against the eligibility criteria.

### Data collection

The following data were collected for each patient: age at diagnosis, sex, tumour site (appendix, caecum, ascending colon, hepatic flexure, transverse colon, splenic flexure, descending colon, sigmoid colon, or rectosigmoid junction), presentation (incidental, screening, symptomatic, or emergency), body mass index (BMI), details of chemotherapy treatment, baseline neutrophil count, baseline lymphocyte count, radiological stage (T and N) at diagnosis, histological subtype (adenocarcinoma or mucinous adenocarcinoma), tumour grade (poorly differentiated or moderate to well differentiated), pathological stage (T, N, and M where appropriate) following surgery, and MMR status. Baseline c-reactive protein (CRP) and platelet count were collected following initial analysis.

Details of radiological staging at diagnosis were obtained from multidisciplinary team (MDT) meeting documentation and CT reports. MDT meetings were attended by Consultant Surgeons, Pathologists, Radiologists, Oncologists, and Nurse Specialists, each with expertise in CC. Details of pathological staging were taken from post-operative pathology reports. All radiological staging was performed by Consultant Gastrointestinal Radiologists specialising in colorectal cancer and pathological staging by Consultant Gastrointestinal Pathologists, with a minimum of 3 years subspeciality practice (range 3–30 years). For patients in this study, radiological staging of lymph nodes was not performed with specific pre-defined criteria. Standardised radiological template reporting was not used; however, a predicted TNM stage and identification of the affected colonic segment were always provided.

Data were collected from the electronic health record system at a large tertiary oncology centre in the UK. Data were anonymised at the point of collection and stored in accordance with GDPR regulations.

### Statistical analysis

Radiological T and N stage were considered correct if the number matched that of pathological staging. Staging subgroups were not considered. Patients who were radiologically staged as Tx were excluded from analysis of T stage accuracy, but included in analysis of N stage. The proportions of correctly staged patients were compared between the dMMR and pMMR groups for both T and N staging separately. These comparisons were also performed with staging assessed as dichotomous groups for more favourable localised *vs* less favourable advanced disease: T1/2 *vs* T3/4 is referred to as “T status” and N0 *vs* N1/2 as “N status”. These categories were chosen as they are used clinically in making treatment decisions, such as the allocation of NAC.^3^

Baseline patient characteristics were explored to identify significant differences between the dMMR and pMMR groups. Any characteristics found to be significantly different were analysed for an association with pathological staging.

GraphPad Prism 8 (GraphPad Software; San Diego, CA) was used to perform statistical analysis. χ^2^ analysis was used to compare staging accuracy between the dMMR and pMMR groups. For T and N “statuses”, the sensitivity, specificity, positive-predictive value (PPV) and negative-predictive value (NPV) of CT were calculated for the dMMR and pMMR groups. T tests were used to compare mean values of normally distributed data between the patient groups. Receiver operating characteristic (ROC) curves were generated to explore associations between patient factors and pathological staging. A *p-*value of < 0.05 was considered the threshold for statistical significance.

## Results

Table 1 details the characteristics of patients in each group. There were 44 patients in the dMMR group and 57 patients in the pMMR group. The eligibility criteria were applied after generating the initial patient data set, which led to uneven groups. Only one patient had non-contrast-enhanced CT. Table 2 summarises the staging data for patients in each study group.

**Table 1.** Patient characteristics

	dMMR (*n* = 44)	pMMR (*n* = 57)	*p-*value
Age at diagnosis, years			
Median	75	73	0.65
Range	23–89	45–90	
Sex, n (%)			0.18
Male	15 (34)	27 (47)	
Female	29 (66)	30 (53)	
BMI, kg/m^2^			
Mean	26.2	27.7	0.21
Range	15.6–50.3	13.1–43.8	
Primary tumour location, n (%)			0.0095
Appendix	1 (2)	0 (0)	
Caecum	12 (27)	14 (25)	
Ascending colon	15 (34)	13 (23)	
Hepatic flexure	3 (7)	0 (0)	
Transverse colon	9 (20)	5 (9)	
Splenic flexure	2 (5)	5 (9)	
Descending colon	0 (0)	2 (4)	
Sigmoid colon	2 (5)	17 (30)	
Rectosigmoid junction	0 (0)	1 (2)	
Side of colon affected, n (%)			<0.001
Right	40 (91)	32 (56)	
Left	4 (9)	25 (44)	
Presentation, n (%)			0.86
Incidental	1 (2)	3 (5)	
Screening	10 (23)	13 (23)	
Symptoms	27 (61)	35 (61)	
Emergency	6 (14)	6 (11)	
Chemotherapy treatment, n (%)			0.029
None	41 (93)	44 (77)	
Adjuvant	3 (7)	13 (23)	
Neutrophils, ×10^9^/L			
Mean (SD)	6.54 (3.32)	5.15 (2.04)	0.012
Range	1.55–17.87	1.19–11.16	
Lymphocytes, ×10^9^/L			
Mean (SD)	1.62 (0.73)	1.53 (0.52)	0.50
Range	0.19–4.00	0.71–3.08	
Neutrophil:lymphocyte ratio			0.13
Mean (SD)	5.63 (8.62)	3.77 (2.14)	
Range	1.19–59.05	0.70–11.52	
Histology, n (%)			0.0045
Adenocarcinoma	27 (61)	49 (86)	
Mucinous adenocarcinoma	17 (39)	8 (14)	
Differentiation, n (%)			0.0063
Well/moderate	29 (66)	52 (91)	
Poor	13 (30)	4 (7)	
Unknown/not applicable^*a*^	2 (5)	1 (2)	
Stage^*b*^, n (%)			0.27
I	11 (25)	12 (21)	
II	21 (48)	21 (37)	
III	12 (27)	21 (37)	
IV	0 (0)	3 (5)	

dMMR = mismatch repair deficient; pMMR = mismatch repair proficient; BMI = body mass index; SD = standard deviation.

a Differentiation classed as ‘not applicable’ for some mucinous tumours, in accordance with current Royal College of Pathologists guidance.^14^

b According to AJCC system v. 8.^13^

**Table 2.** Radiological and pathological staging data

dMMR (*n* = 44)				pMMR (*n* = 57)			
Radiological		Pathological		Radiological		Pathological	
**Tx**	3			**Tx**	4		
**T1**	0	**T1**	5	**T1**	0	**T1**	6
**T2**	10	**T2**	6	**T2**	12	**T2**	10
**T3**	19	**T3**	23	**T3**	29	**T3**	25
**T4**	12	**T4**	10	**T4**	12	**T4**	16
**N0**	16	**N0**	32	**N0**	29	**N0**	33
**N1**	21	**N1**	10	**N1**	20	**N1**	17
**N2**	7	**N2**	2	**N2**	8	**N2**	7

dMMR = mismatch repair deficient; pMMR = mismatch repair proficient.

Staging performed according to AJCC system v. 8.^13^

### Accuracy of T staging

Individual T stage was correctly predicted in 25 of 41 dMMR (61%) and 29 of 53 pMMR (55%) tumours. Three patients in the dMMR group and four in the pMMR group were radiologically staged as Tx (Table 2) and were therefore not included in this part of the analysis. Of the tumours that were incorrectly staged, overstaging occurred in 10 of 16 dMMR (63%) and 13 of 24 pMMR (54%) tumours. There was no significant difference in the proportions of correctly staged or overstaged patients between the dMMR and pMMR groups (*p* = 0.54 and *p* = 0.60, respectively).

“T status” was correctly predicted in 35 of 41 dMMR (85%) and 47 of 53 pMMR (89%) tumours. There was no significant difference between these proportions (*p* = 0.63). The relative lack of patients with incorrect “T status” precluded further analysis of under- or overstaging. The sensitivity, specificity, PPV and NPV for predicting “T status” in the dMMR group were 88%, 75%, 94% and 60%, respectively, and 95%, 71%, 90% and 83%, respectively, in the pMMR group.

### Accuracy of N staging

**Figure 1.**
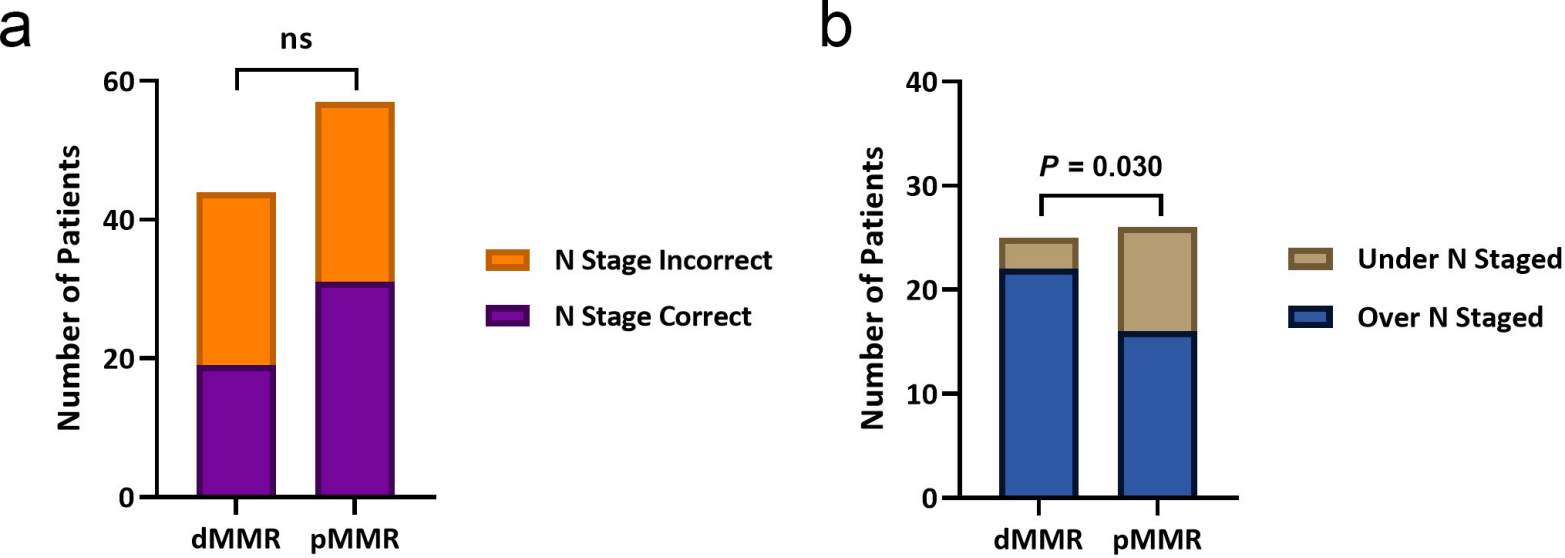
Accuracy of predicting lymph node stage. (a) There was no significant difference in the proportion of patients with correctly predicted N stage between the dMMR and pMMR groups (*p* = 0.26). (b) However, of the patients with incorrect N stage, a significantly greater proportion were overstaged in the dMMR group, compared with the PMMR group (*p* = 0.030). dMMR, mismatch repair deficient; ns, not significant; pMMR, mismatch repair proficient.

N stage was correctly predicted in 19 of 44 (43%) and 31 of 57 (54%) patients in the dMMR and pMMR groups, respectively. Of the cases with incorrect N stage, 22 of 25 (88%) and 16 of 26 (62%) were overstaged in the dMMR and pMMR groups, respectively. Whilst there was no significant difference in N staging accuracy between the groups (*p* = 0.26), incorrectly staged patients in the dMMR group were significantly more likely to be overstaged, compared to those in the pMMR group (*X* ^2^(1, *n* = 51) = 4.70, *p* = 0.030) (Figure 1).

**Figure 2.**
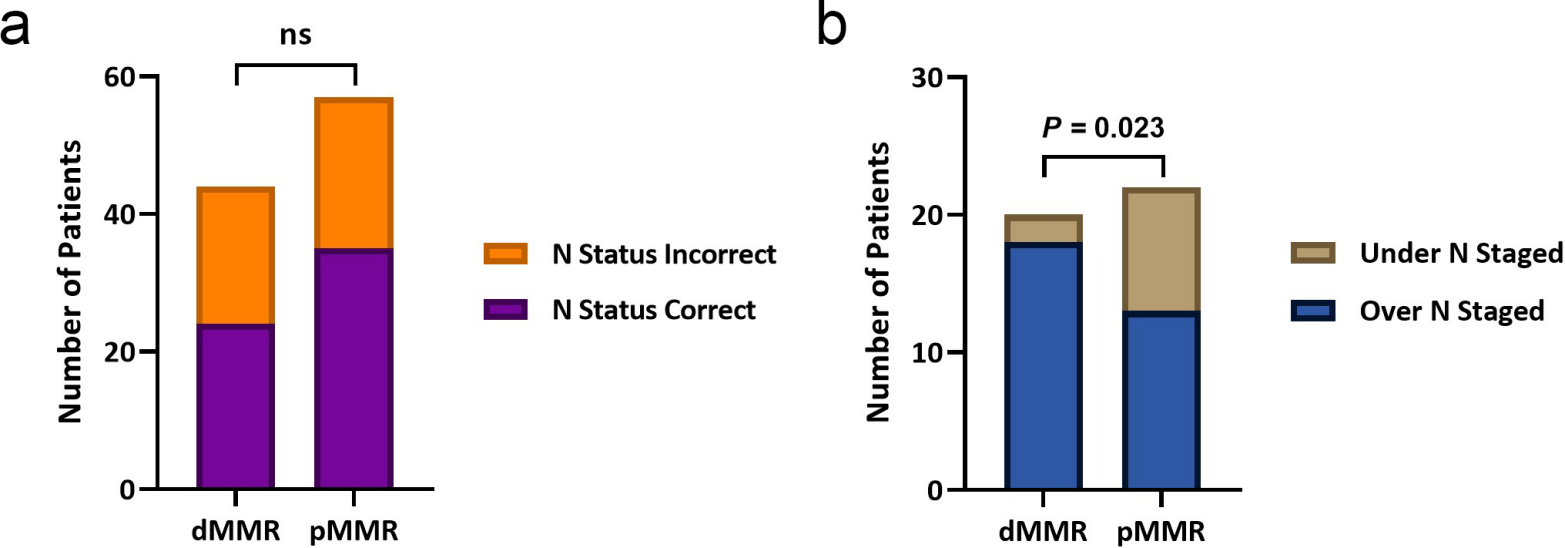
Accuracy of predicting lymph node “status”. (a) There was no significant difference in the proportion of patients with correctly predicted “N status” between the dMMR and pMMR groups (*p* = 0.49). (b) However, of the patients with an incorrect “N status”, a significantly greater proportion were over-staged in the dMMR group, compared to the pMMR group (*p* = 0.023). dMMR, mismatch repair deficient; ns, not significant; pMMR, mismatch repair proficient.

“N status” was correctly predicted in 24 of 44 (55%) and 35 of 57 (61%) patients in the dMMR and pMMR groups, respectively. Of the cases with an incorrect “N status”, 18 of 20 (90%) and 13 of 22 (59%) were overstaged in the dMMR and pMMR groups, respectively. There was no significant difference in the accuracy of predicting “N status” between the groups (*p* = 0.49). However, the proportion of patients whose “N status” was overstaged was significantly greater in the dMMR group, compared to the pMMR group, (*X* ^2^(1, *n* = 42) = 5.18, *p* = 0.023) (Figure 2). The sensitivity, specificity, PPV and NPV for determining “N status” in the dMMR group were 83%, 44%, 36% and 88%, respectively, whereas in the pMMR group they were 63%, 61%, 54% and 69%, respectively.

### Patient characteristics to support staging

**Figure 3.**
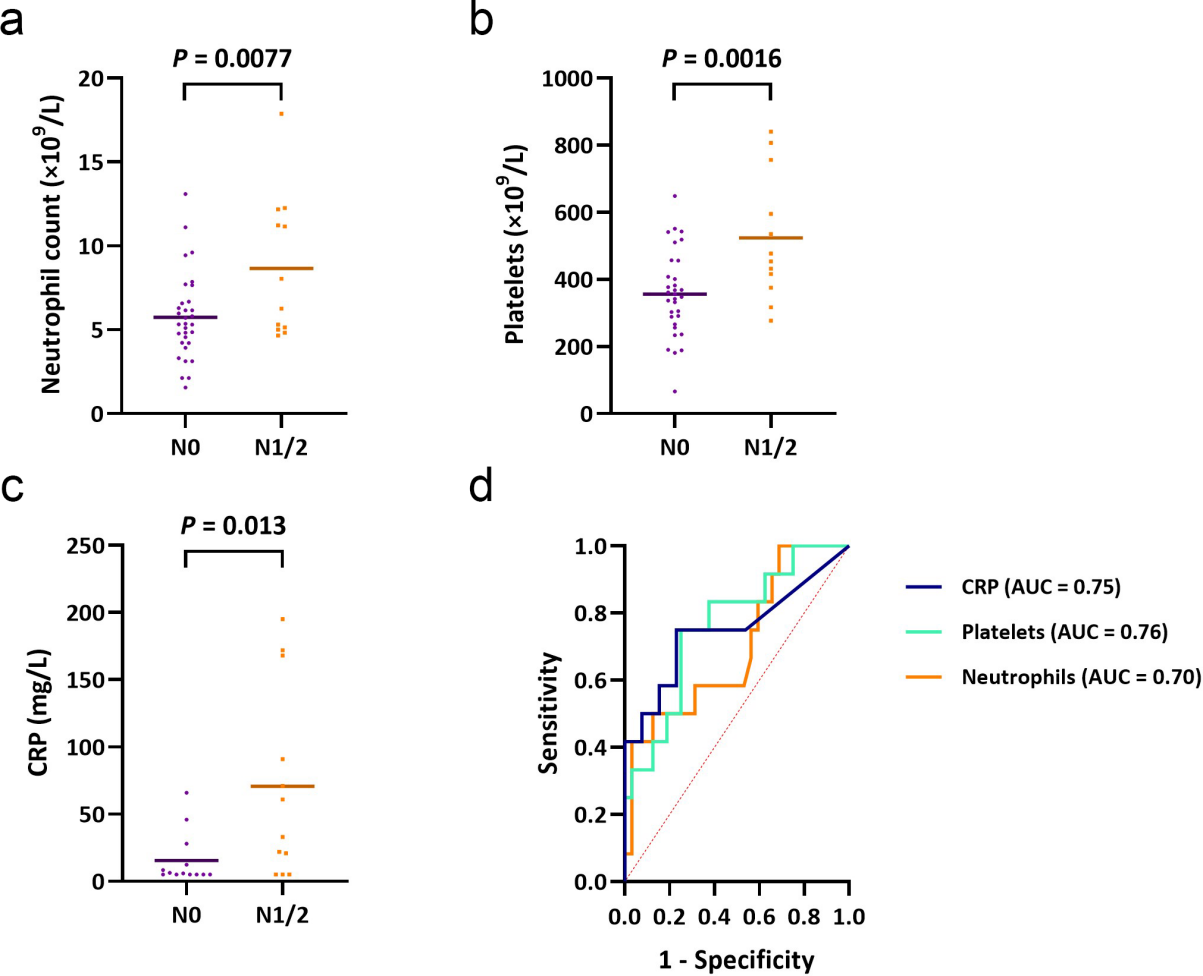
Inflammatory markers and pathological lymph node “status” in dMMR colon cancer. (a) Mean baseline neutrophil count was significantly higher in dMMR colon cancer with N1/2 “status”, compared to N0 “status” (*p* = 0.0077). (b) Mean baseline platelet count was significantly higher in dMMR colon cancer with N1/2 “status”, compared to N0 “status” (*p* = 0.0016). (c) Mean baseline CRP was significantly higher in dMMR colon cancer with N1/2 “status”, compared to N0 “status” (*p* = 0.013). (d) ROC curve analysis resulted in an AUC of 0.70 (*p* = 0.044), 0.76 (*p* = 0.0078), and 0.75 (*p* = 0.034), for neutrophil count, platelet count and CRP, respectively. AUC, area under the curve; CRP, c-reactive protein; dMMR, mismatch repair deficient.

Of the characteristics that differed significantly between the dMMR and pMMR groups (Table 1), neutrophil count and tumour grade were also associated with pathological “N status” in dMMR CC. Mean neutrophil count at diagnosis was significantly higher in dMMR CC with pathological N1/2 “status” than with N0 “status” (8.66 × 10^9^  L^−1^ *vs* 5.74 × 10^9^  L^−1^; *p* = 0.0077) (Figure 3a). Mean platelet count and CRP were also both significantly higher in dMMR CC with pathological N1/2 “status” than those with N0 “status” (523 × 10^9^  L^−1^ *vs* 357 × 10^9^  L^−1^; *p* = 0.0016 and 70.75 mg L^−1^ *vs* 15.62 mg L^−1^; *p* = 0.013, respectively) (Figure 3b and c). ROC curve analysis to assess the predictive value of these inflammatory markers provided an area under the curve (AUC) of 0.70 (*p* = 0.044), 0.76 (*p* = 0.0078), and 0.75 (*p* = 0.034), for neutrophil count, platelet count and CRP, respectively (Figure 3d).

A significant association was also seen between tumour grade and pathological “N status” in dMMR CC, with poorly differentiated grade more common in those with N1/2 “status” (*X^2^* (2, *n* = 44) = 11.1, *p* = 0.0039). No association was seen between histological subtype and “N status” in dMMR CC (*p* = 0.58). None of the positive associations were seen in pMMR CC (*p* > 0.05 for all).

## Discussion

In this study, we have demonstrated a novel relationship between markers of systemic inflammation (platelet count, CRP and neutrophil count) and the presence of lymph node metastases in dMMR CC. Patients with dMMR CC were also found to have a significantly greater risk of N overstaging, compared to those with pMMR CC. Using routine blood tests to assess systemic inflammation has the potential to support and improve the accuracy of radiological staging in dMMR CC.

The characteristics of patients in our study are in keeping with the wider CC population, including those specific to dMMR CC, such as right-sided location, mucinous histology, poorly differentiated grade, and higher neutrophil count.^12,15^ Likewise, pathological staging data were also consistent with the known differences between dMMR and pMMR CC, with T3/4 “status” more common and N1/2 “status” less common in the dMMR group (Table 2).^12^ We found no difference in the accuracy of radiological T stage or “T status” between dMMR and pMMR CC, or in the proportion of tumours which were under- or overstaged. CT is known to have a high level of accuracy for predicting “T status” in CC, which is of clinical importance given its role in selecting patients to benefit from NAC; the FOxTROT study found significant benefit with NAC in patients with radiologically staged T3/4 CC^3^. Nevertheless, once NAC is implemented as a routine treatment, further improvements in the accuracy of predicting “T status” will be crucial to prevent overtreatment of patients who are unlikely to derive benefit. Notably, FOxTROT failed to show definitive benefit for NAC in dMMR CC. However, there is growing evidence for the use of neoadjuvant immunotherapy in dMMR CC,^16^ which may rely on accurate radiological staging to stratify patients effectively. The overall sensitivity of CT for predicting “T status” in our cohort exceeded that reported in a recent Danish study (92% *vs* 68%).^17^ Possible reasons for this include: the staging of patients in our study was performed exclusively by expert Gastrointestinal Radiologists and Pathologists, while in the previous study these details were unclear; a complete radiological staging data set from routine clinical practice was provided in our cohort, whereas staging details were missing in approximately 15% of patients in the prior study; and finally, patients were taken from two different geographical populations and healthcare systems.

Whilst no significant difference was found in the accuracy of N stage or “N status” between the dMMR and pMMR groups, patients with dMMR CC were significantly more likely to be N overstaged than those in the pMMR group. The clinical implications of N overstaging may include more radical surgery (*e.g.* central vascular ligation) with greater morbidity^18–20^ and inappropriate chemotherapy planning. With increasing evidence for the use of neoadjuvant chemotherapy (pMMR CC)^3^ and immunotherapy (pMMR and dMMR CC),^16^ and the refinement of criteria for patient selection, accurate N staging may become even more important. Overall, we achieved a greater sensitivity (69% *vs* 55%) but lower specificity (52% *vs* 66%) for determining “N status” using CT than a recent study, which we believe is most likely a consequence of having a greater proportion of dMMR CC (44% *vs* 24%) and associated inflammatory lymphadenopathy in our study.^17^ Furthermore, N staging may also have been performed in many of these patients using strict size criteria, which have been shown to be insufficient for reliably identifying lymph node metastases.^6,7^ A recent study highlighted significant differences in the radiological appearance of the primary tumour and lymph nodes between dMMR and pMMR CC.^21^ This study also illustrated that the features most predictive of lymph node metastases differed according to MMR status, with largest short axis diameter and node heterogeneity being most effective for dMMR and pMMR CC, respectively. Assessing lymph nodes in an MMR status-agnostic manner is therefore insufficient and may have contributed to the findings in our study. The lack of a significant difference in overall N staging accuracy between the dMMR and pMMR groups was surprising. We expected N staging accuracy to be significantly lower in those with dMMR CC, compared to pMMR CC, due to the presence of enlarged inflammatory lymph nodes. Inconsistencies in staging accuracy between studies may reflect the challenge of radiological N staging in clinical practice and the variation that likely exists between radiologists and centres. Beyond CC, radiological N staging accuracy has not been explored in other dMMR cancers and may represent a valuable avenue of future research.

The systemic inflammation seen in MMR deficiency results in the development of enlarged reactive lymph nodes that may mimic nodal metastases on CT. Therefore, patients with dMMR CC are inherently at risk of N overstaging, whether they have nodal metastases or not. We observed higher neutrophil count, platelet count, and CRP in dMMR CC with nodal metastases, suggesting that more extensive disease may stimulate additional systemic inflammatory pathways, compared with localised and possibly more indolent disease. To our knowledge, this study is the first to report such a relationship, and should now be validated prospectively in larger clinical trials. Patients with dMMR CC and N1/2 “status” were also more likely to have a poorly differentiated tumour, compared to those with N0 “status”. Identifying additional features associated with nodal metastases in dMMR CC would facilitate the development of a model that integrates radiological and biochemical data to improve staging accuracy. For example, a composite score of neutrophil count, platelet count and CRP may hold value for effectively downstaging patients with dMMR CC and radiological N1/2 “status”. However, such approaches rely on the knowledge of MMR status at diagnosis, which remains inconsistent in clinical practice.^22^

Our study is limited by retrospective data collection and the inclusion of patients from a short time period. Furthermore, relying on historical documentation may have introduced information bias, while missing data may limit patient eligibility and risk selection bias. Our analysis of T staging accuracy was restricted to whole numbers only and not staging subgroups. Whilst the subdivision of T3 tumours into those that are T3a/b or T3c/d holds prognostic significance,^23^ we were unable to make this distinction with a small sample size. Finally, the lack of specific N staging criteria in our centre may limit our understanding of how MMR status impacts N staging elsewhere. Overall, these issues may limit the generalisability of our findings to clinical practice. However, we have addressed some of the limitations of previous research by providing data on UK patients, compiling a data set with complete staging and MMR status for all patients, and utilising staging performed by expert Gastrointestinal Radiologists and Pathologists.

## Conclusion

In conclusion, whilst the accuracy of radiological T and N staging is similar between dMMR and pMMR CC, patients with incorrect N stage in dMMR CC are more likely to be overstaged. Baseline inflammatory markers and tumour grade are associated with the presence of nodal metastases in dMMR CC and may play a role in improving pre-treatment clinical N staging. Our data suggest that MMR status should be considered during MDT evaluation of radiological staging for all patients with newly diagnosed CC, especially with the increasing role for neoadjuvant therapies and more aggressive mesocolic surgical resection. Further research into the association between the host inflammatory response and the presence of lymph node metastases in dMMR CC is warranted.
